# Dentition and Its Derangements

**Published:** 1861-08

**Authors:** A. Jacobi


### Selections.

Dentition and its Derangements.—By *A. Jacobi, M. D.* —*Lecture IV.*—In order to complete the anatomical and physiological part of our subject, let me proceed to some remarks on the permanent teeth. You remember, from my previous lecture, what I said, following the description of the process as given by Harrison, on the first formation and development of the temporary ones. Soon after the commencement of the saccular stage of the deciduous teeth, the rudiments of the second or permanent set are developed. About the fourteenth week of fœtal life, the deep portion of the primitive dental groove is closed in, and contains the sac and papillæ of the ten milk teeth; the upper or superficial portion of the groove remains open, and is then named *secondary dental groove.* In this commence the rudiments of the permanent teeth. At first a small depression is observable behind the superior openings of the milk sacs; this increases, and forms the *cavity of reserve.* These cavities are lined by an inflection of the mucous membrane, and at the bottom of each a small papilla is formed; they gradually recede from the surface, and are thereby converted into follicles, and finally into closed sacs, which lie to the inner side of, and in close contact with, the former set, and inclosed in the same submucous tissue. The necks of these sacs, by which they originally communicated with the general mucous membrane, continue as obliterated cords leading to the surface of the gum, internal to the deciduous teeth. These cords have been named the *gubernacula*, or *itinera dentium*, roads of the teeth, without having, however, any such office as the name would appear to imply. The primitive dental groove, behind the posterior deciduous molar, does not close so soon as the anterior portion, and in it are developed, about the fifth month of fœtal life, the follicle and papilla of the anterior permanent molar. After its follicle has closed, the dental groove closes over it, leaving a space between the gum and the sac of this tooth; in this is a cavity of reserve of mucous membrane for the second permanent molar, and one also for the third molar or wisdom tooth. As the deciduous sacs, as well as the anterior permanent ones, increase in size more rapidly than the bones can elongate, this cavity for the permanent molars is pressed backwards into the maxillary tuberosity above, and into the root of the coronal process below; but in a few months after birth, as the jaws increase in size and length, the first permanent molar returns to its proper level in the dental range; the cavity of reserve behind them dilates into the space the first molar occupied, and in it is developed the papilla for the second permanent molar. In the course of time, as the jaws further elongate, this tooth advances and descends, and the remainder of the cavity of reserve dilates behind for the third permanent molar or wisdom tooth.

The permanent sacs at first receive their vessels from those of the gum, but afterwards from the temporary sacs; and as they retire into their own cells, these new vessels enter into new dental canals, which become permanent. In the course of a few years, and after all the temporary teeth have appeared, the further elongation of the jaws admits of space for the first true molar to protrude; this usually occurs between the sixth and eighth years, and sometimes even sooner. At this age there are fifty-two teeth in the two jawbones, viz: twenty deciduous teeth, twenty permanent beneath these, and the twelve posterior molars; and when all the anterior permanent teeth have become enlarged, they press upon the anterior wall of their alveoli, which soon undergo absorption; and then each tooth comes a little forwards into the lower part of the alveolus of the milk tooth; the fangs of the latter are absorbed and gradually wholly removed, and then the crown falls out of the sac, and the permanent tooth supplies its place.

The cause of the disappearance of the roots of the temporary teeth is sought for in the loss of nutrition from the pres sure of the *subjacent* permanent tooth, and perhaps in con temporaneous increase in the general injection and nutrition, bringing on liquefaction and absorption. Some pressure is necessary, at all events, for whenever there is no pressure from below, the temporary tooth is not removed. But you must not imagine that the permanent tooth exercises any immediate pressure on the blood vessels, thereby depriving the temporary tooth of its nutrition ; if this was the case, the permanent tooth would exercise just the same influence, at a much earlier period, even while the temporary itself was not fully developed.

The crowns of the permanent molar teeth, further, are perfectly unable to exercise any pressure on the blood-vessels of the temporary, as they are situated between their roots. The nutrition of the temporary teeth is impaired by two facts, first by the increasing development of the permanent themselves, and, further, by the development of the maxillary bones, which contract and partially obstruct the canals through which the branches of the maxillary artery penetrate to the tooth. The pressure of the permanent tooth on the temporary one is not at all direct; nor is it necessary that it should be so.

Nature usually, in building up and destroying, works very slowly and invisibly. A fine instance of what a slight pressure for a protracted period may effect, and how bones are absorbed from the pressure of a slight physical influence, is given in the fact, that aneurisms of large arteries at some parts of the body, where they are in the neighborhood of bones, destroy the bone by slow, gradual absorption. Thus aneurisms of the aorta are reported to have produced absorption of part of some spinal vertebræ, and I have myself seen two or three costal cartilages absorbed from the constant hammering against the chest by a large aneurism of the ascending aorta.

You see, therefore, that the pressure of the permanent tooth inclosed in its cell, on the wall separating it from the temporary tooth, it being slowly and continually forced upwards, may be deemed sufficient to bring the root of the temporary tooth to absorption. The effect of the permanent teeth is not in one direction only, for you know that the permanent teeth are not situated in the same horizontal line ; the steady slow pressure is exercised upwards and laterally, thus the roots of the molar teeth are absorbed on their inner sides, and the middle permanent incisors press not only on the corresponding temporary, but the lateral ones also. The root of the temporary tooth, while being deprived of its normal nutrition by pressure exercised on the periosteum, is liquefied by the increased action in the surrounding parts, brought under the influence of the numerous absorbent vessels contained in the sac of the onward growing tooth, and excreted like so many other effete matters.

The vessels rendering this service to the organism have been made the subject of particular study by Boardet, who called them "appareil dissolvant," and Delabarre, that learned humbug and nostrum-seller, who comprehends them under the name of "appareil absorbant." This resorption can take place as long as the root is in some connection with the surrounding parts. If it ceases to be so, the vital powers of absorption are replaced by another; in this case the root has the general effect of a foreign body brought by some means or other into contact with and imbedded in the organism, to produce inflammation and to be removed by suppuration. Thus no resorption takes place even when the crown of the permanent tooth comes into immediate contact with the root of the temporary; in which cases the temporary teeth, particularly the molar, are found to be turned over and produce, by the effect of their sharp roots, deep ulcerations in the cheek, which will not heal before the temporary tooth is removed.

The permanent teeth appear no more nor less at regular periods than the temporary ones. About the seventh year, or earlier, as I mentioned before, the first permanent molar appears, nearly about the time when the first temporary incisors are replaced by the permanent. After all the incisors are changed, the anterior and posterior temporary molars are successively shed and replaced by the permanent bicuspids; the canines are changed about the tenth or eleventh year. About the twelfth or thirteenth the second permanent molars appear; the last molars, or wisdom teeth, usually some time between the twentieth and thirtieth.

Ossification requires but a short time in the deciduous teeth and longer in the permanent. A permanent incisor requires seven years, a canine twelve, a molar from eight to ten years. Ossification commences at the very same time in incisors and the first molars, as is proved by the dissection of the jawbones of infants who died in the first months after birth. It progresses more rapidly in the female than in the male sex; girls, therefore, have their permanent teeth sooner than boys.

In the lower jaw of a child three years of age, the permanent teeth are still in an oblique direction. Only the middle incisors, which are the highest, are in a nearly vertical position; the lateral incisors are situated more inwards, and more obliquely; the lowest are the canine teeth. Higher, and between the roots, we find the molar teeth in their first stages of development, or rather the first one; for as to the second, we find nothing but the cells in which it will be contained in future. The time of its first formation is about the fifth year. As it requires about eight years for its complete ossification, it makes its appearance about the thirteenth year of life. The commencement of ossification in the third molar tooth, and particularly its appearance, is more uncertain, as it depends on local circumstances. It does not usually appear before the twentieth year, but in some cases, according to C. Harris, does not show itself, until the thirtieth or even fortieth year, and Canton extracted one for a gentleman seventy-four years of age, who informed him that it was not out until he had attained his seventieth year.

The maxillary bones of a child of from four to five years contain so many and so large cells for both the temporary and permanent teeth, that but very thin osseous walls form a bridge between the external and internal wall of the jawbones. Nevertheless, every tooth, both temporary and permanent, receives a ramification from the common maxillary blood-vessels and nerve. There is sometimes, according to Delabarre's observation, an anomaly in the lower jaw, of this sort, that the submaxillary artery and nerve, right at their entrance into the lower maxilla, divide into two branches, one of which feeds the temporary, the other the permanent teeth. The periosteum of the alveolar cell, being a mere continuation of the external periosteum, takes the blood-vessels from the maxillary artery, branches of which penetrate the porous osseous substance.

A very interesting subject relating to our investigations is that of the so-called third dentition. Is there at all a third dentition? Are those teeth which we are used to call permanent, not permanent, but subject to be temporary only in proportion to those which are to be as it were more permanent? Certainly there are a number of cases reported, in which the teeth are said to have fallen out twice, and to be replaced twice. There is one case even of the following description:—In a girl the first replacement, the second dentition, took place at six years, the third dentition at twelve; this latter was complete in a single year. This case, our author says, "is highly interesting for two reasons, first, because it occurred in a young individual, while cases of third dentition have been hitherto related of old people only; second, because all the teeth were replaced here by others, while the third dentition has always been incomplete, and limited to the appearance of two or three teeth only." The case, gentlemen, looks so very interesting and beautiful, that I am afraid the reporter is greatly mistaken, or has been grossly imposed upon. Other cases of third dentition are reported, but scarcely any of such a remarkable kind as this case. At all events we require a good deal of belief in the veracity or the judgment of a writer, if we are to take as scientific facts such reports as are in open conflict with the known laws of anatomy and physiology. W. Jackson has the cases of a man of sixty-four, and of a woman of eighty years, in whom a third set of incisors was observed; in one of them the old teeth had just fallen out to make room for the new ones. Sorgoni reports the case of a boy exhibiting a third dentition before he was twelve years of age, and Andral has collected from literature twelve cases of the same anomaly. Lison reports the case of a boy, Eugene Cavillan, thirteen years old, of young and healthy parents, of good constitution and well, and without any anomaly in his general development. The second dentition took place when he was nine years old. Soon after, all his twenty-eight teeth were replaced by others; the same occurrence took place between his tenth and eleventh year, and again between the eleventh and twelfth. When the case was reported by the author, the boy was said to be in his thirteenth year; at this age a new set of teeth was being developed; the first inferior molar teeth of the right side fell out, to give way to another that was already visible. The teeth that had fallen out had no roots, which appear to be eroded. The removal and replacement took place always in the usual order, the teeth being small, white, and of normal shape and position. The gums were red and somewhat tumefied, and the general health of the boy satisfactory.

I consider it a characteristic occurrence that curiosities like those alluded to are more numerous in old, very old books, than in modern ones. Storch, alias Pelargus, who wrote in 1750, reports the case of a lady of seventy years, who, after having lost all her teeth for a number of years, had a new incisor at that advanced age. He further has the case of his own daughter, who cut five molar teeth in her twentieth year; lost them all, and had new ones in their place when she was thirty-eight years old. Before this time, our author says, the lady was always sick from this abnormal teething—the symptoms enumerated, however, being evidently of uterine and hysteric nature; but after the last teeth cut, she became healthy, and strong, and fat. Old Paulinus relates the case of a Countess of Detmond, who lived up to a third dentition, in 1589, and grew one hundred and forty years old. The younger Pliny has the observation of the last molar tooth appearing at eighty years of life; Schottus at forty years, in a physician of his acquaintance; Cardamus, the celebrated mathematician and inventor of the Cardamian Formula, is reported to have cut a tooth at forty-three; several soldiers at forty-three, forty four, and forty-five; several others, according to Sennertus, at sixty-three, seventy-five, eighty, eighty-one, eighty-eight, even at one hundred and four years of age. In an old book of 1725, there is the case of a woman of sixty-six years, who got not only new teeth, but new brown hair, instead of her former grey. Johannes G. Slevogt reports, in 1733, the case of a captain who cut new teeth at ninety-four years of age, and died soon afterwards; we do not learn whether the old man died in consequence of teething, or whether, if he had not teethed again, he would not perhaps have lived up to our times, and been still older than ninety-four. But the greatest curiosity I have ever been able to hunt up is the following, reported by Möllenbroc, a century and a half ago. There lived at Leipsic a noble lady who had five children; with every confinement she cut a molar tooth. As soon as one of her new teeth got loose, the child who was born at the time when it was cut, was affected with some severe disease. If such a tooth fell out, she was always certain that the corresponding child was surely going to die. And so it happened, adds our honest author, all the three children died before the mother. Thus you perceive, gentlemen, that as it is said to be customary nowadays that children die from their own teething, it was customary for children in olden times to perish from the dental troubles of their mothers.

Both Courtois and Aimonino have published cases in which a third dentition took place after the permanent teeth fell out; Courtois is of the opinion that the third dentition is observed in the incisors only. If such was the case, why, we must expect that the teeth of the third period were formed contemporaneously with those which were then eliminated by the growth and onward pressure of the subjacent ones. At all events, the belief in a third dentition was so general formerly, that decayed teeth would be removed in the hope that a replacement would take place. Professor Nessel, whose name I have mentioned before, has observed a girl whose middle upper incisors had been extracted in the hope that they would reappear. But not only no new teeth appeared, but the space in which two teeth had been seated formerly was so much intruded upon by the neighboring ones, that but one artificial tooth found sufficient room afterwards. Now, what nature will not do in youth, she will hardly succeed in doing in old age, where all the reported cases of third dentition are said to have occurred. There is less probability of new germs of teeth forming and developing themselves in advanced life, than that there have been from the beginning supernumerary teeth; instances of which have been reported by Roysch, besides those enumerated in my second lecture.*

The fact that teeth will protrude, sometimes, at old age, is undoubtedly true. Instead of being, however, the symptoms of a renewed power of reproduction, they are, in Professor Nessel's opinion, frequently the results of regressive life; as they become visible after the diminution of the alveoli, and the decrease of the thickness of the gums. Such teeth were always formed, but were either invisible from being sometimes incuneated like the canine, or from being covered by an osseous mass, like the wisdom teeth. The second molar tooth, particularly, has been observed to reappear in advanced age, but only after the temporary second molar had kept its place, and fallen out at a very advanced period of life. It is not a very rare occurrence that the temporary second molar tooth remains at its place up to the fortieth year, and thus there can be no mystery nor wonder about the fact that another tooth will make its appearance afterwards.

The temporary second molar tooth, however, is not the only one that will remain for a long period, and thereby retard the second dentition. Linderer reports the case of a girl who had her first permanent molar tooth with her eighth year, but whose second dentition did not begin before the fifteenth year. Another healthy and robust girl of fourteen years, who never had the four upper incisors, had all her other milk teeth, yet without there being any probability of an approaching change. Murat has the case of a robust young man of

---

* American Medical Times, 1860, p. 419.

seventeen, who had all his milk teeth but five; and Bird and Maingault report similar cases. Other cases are noticed, by Linderer, of single milk teeth remaining up to the thirtieth or fortieth year; and Riecken gives the history of a man of eighty-five, who cut a number of incisors and molars, and is said to have suffered during his cutting a molar in his left lower jaw, from cerebral congestion, which was relieved, after local depletion had no effect whatever, by spontaneous hemorrhage from the inner angle of the eye. Finally, a woman of forty-three years was observed by Düntzer, who had all her milk teeth left. After she had been suffering from intense pain in her head and upper jaws, from swellings of the gums, and diarrhea, four teeth protruded behind the upper incisors; they were smaller and sharper, and troubled the functions of both mastication and articulation. After the lapse of a year, the same symptoms were observed in the right lower maxilla, which never had any molars before.

Kneissel reports the case of a lady who reproduced four inferior incisors in her fifty-fourth year, after having worn artificial teeth for some time; and a right upper incisor, in place of one that had just fallen out, two years afterwards. The teeth which had fallen out were undoubtedly the temporary ones that had never been removed, and finally fell out at an advanced age from being either pressed upwards mechanically, or being decayed; nobody can say which, as the report does not contain anything beyond the facts I have related. Professor Hessel has the case of a lady who cut a fine white canine tooth at fifty years of age. This tooth became more visible from year to year, not because of its growth, but because of the decrease of the alveolar margins of the maxillary bone. It had been, in his opinion, always formed and ready to protrude, and would have done so but for the other teeth occupying the space naturally designed for it. The same author reports in his book on dentistry (1856) the case of a gentleman of thirty years, who still had his temporary upper incisors.—*American Medical Times.*

ALUMINUM IN GREENLAND.—The Edinburgh *Courant* states that two Danish vessels have sailed from Leith for Greenland, for procuring cargoes of cryolite—the mineral from which aluminum is obtained in largest quantities. Several very valuable minerals are obtained from Greenland. Plumbago is abundant in these regions; but the cryolite is the most important of Greenland's products, because aluminum is daily increasing in favor, as a most beautiful metal, capable of superseding silver for many purposes.

---

A GENTLEMAN in Higginsport, Ohio, writes to the *Journal of Materia Medica* that he has used the pills of hydrocyanite of iron for epilepsy for several months past, and that he has had but one convulsion since, which occurred about two weeks after he first commenced. He has since been free from any symptoms of their occurrence.

---

NEW SILVER ALLOY.—A beautiful new alloy is stated by foreign contemporaries to have been invented recently, after many experiments, by Messrs. De Ruols and De Fontenay, France. It is said to be well adapted for small coins and industrial purposes. It consists of one-third silver united with 25 to 30 per cent. of nickol, and from 37 to 42 of copper. Phosphorus is used as a flux in making the metals combine, but when first made and cooled, it is very brittle. To render it ductile, the phosphorus must all be removed by reheating, after which the alloy resembles a simple metal, and presents in a very high degree the qualities to which the precious metals owe their superiority. It resembles platina and silver of $\frac{890}{1000}$ in color; it takes a very brilliant polish. Its tenacity and hardness are extreme. It is ductile, malleable, and very difficult of fusion; very sonorous, unalterable in the air, and attacked only by the most energetic re-agents. It has no odor, and its specific gravity is but little inferior to that of silver. It is easy to estimate the important part such an alloy is calculated to play iu the industrial arts, and especially in the silversmith's art—in, to a great extent, replacing silver, of which its price is 40 per cent. less, and as its hardness gives it a marked superiority. Again, articles which are merely silvered or gilt have, it is true, a great advantage in their low price; but they quickly deteriorate, and can be re-silvered or re-gilt only a very few times, after which they must be replaced by new ones, and, in the long run, entail such an outlay as to confirm the old adage, that "the cheapest is the dearest in the end."

